# Healthcare reform and productivity of Hospital: a DEA-based analysis from South West of Iran

**DOI:** 10.1186/s12962-022-00403-x

**Published:** 2023-04-24

**Authors:** Sajad Vahedi, Mansour Zahiri, Narges Pirani, Amin Torabipour

**Affiliations:** 1 Bureau for Health and Social Welfare, Deputy for Scientific, Cultural and Social Affairs, Plan and Budget Organization of the Islamic Republic of Iran, Tehran, Iran; 2 Department of Health Services Management, School of Health, Ahvaz Jundishapur University of Medical Sciences, Ahvaz, Iran; 3 School of Health Management and Information Sciences, Iran University of Medical Sciences, Tehran, Iran; 4 Social Determinants of Health Research Center, Ahvaz Jundishapur University of Medical Sciences, Ahvaz, Iran

**Keywords:** Organizational productivity, Public hospitals, Health resources, Iran

## Abstract

**Background:**

Different healthcare reforms could affect the productivity of hospitals. The aim of this study was to track hospital productivity before and after the recent Iranian healthcare reform in Khuzestan province, South West of Iran.

**Methods:**

Hospital productivity was evaluated through data envelopment analysis (DEA) and Malmquist productivity index (MPI) from 2011 to 2015 for 17 Iranian public hospitals before and after the health sector transformation plan. We assumed an output-oriented model with variable returns to scale (VRS) to estimate the productivity and efficiency of each hospital. The DEAP V.2.1 software was used for data analysis.

**Results:**

After the transformation plan, the averages of technical efficiency, managerial efficiency and scale efficiency in the studied hospitals had negative changes, but technology efficiency had positive changes.44.4% of general hospitals, 25% of multi-specialized hospitals, and 100% of specialized hospitals had positive productivity changes after implementing the health sector evolution plan. The Malmquist productivity index (MPI) had low positive changes from 2013 to 2016 (MPI = 0.13 out of 1) but the mean productivity score had no change after the health sector evolution plan.

**Conclusions:**

The total productivity before and after the health sector evolution plan had no change in Khuzestan province. This and the increase in the utilization of impatient services seemed to be a sign of good performance. But apart from technology efficiency, other efficiency indices had negative changes. It is suggested that in health reforms in Iran, more attention should be paid to the allocation of resources in the hospital.

## Introduction

Continuously rising health care costs and inadequate access of vulnerable populations to needed health care services are persuading health care authorities to implement health care reforms to increase health sector equity and sustainability [1]. These reforms may change the rules of the game in the health sector and affect the productivity of health organizations such as hospitals. [2]. The hospital is an important component of the health system that can absorb majority of health care resources, especially in low- and middle-income countries [3]. Hence, hospital productivity could be considered as the key predictor of the productivity of the health system as a whole.

The Iranian health system is based on the public services provision model. The ministry of health finances and delivers primary healthcare while secondary and tertiary care is financed through insurance schemes and private sectors [4]. In 2016, there were 981 hospitals in Iran, 614 of which were owned by the Ministry of Health and Medical Education (MOHME) [5]. Low bed occupancy and low turnover rate were reported as key problems in the hospitals of Iran [3]. In 2014, the Iranian Health Sector Evolution Plan (HSEP) was devised and implemented by MOHME to increase the coverage of basic health insurance, quality of hospital care and primary healthcare, and reduce out-of-pocket (OOP) payments for inpatient hospital services [6]. This reform increased the demand especially for inpatient health services [7] and also seemed to increase health spending in the country. Hospital care is costly and is believed to be provided with low productivity due to the imbalance between its inputs and outputs in Iran. This issue has created many discussions regarding the better allocation of resources, reducing costs and increasing the efficiency of hospitals [8]. Efficiency and productivity have a well-established association, and to increase hospital productivity, efficiency should be increased [9]. Hence, productivity and efficiency assessment can help identify the performance challenges of hospitals, especially in the dynamic circumstances induced by healthcare reforms [2, 10]. There are several techniques to assess hospital efficiency and productivity. One of the applied and useful techniques is data envelopment analysis (DEA) used to assess hospital productivity in many studies in different countries [10, 11]. In Iran, a number of studies were conducted to assess hospital efficiency and productivity. Kiadaliri et al. showed that twenty nine studies were conducted in the field of efficiency and productivity of hospitals from 2000 to 2012 [12]. Another study also reported a decrease in productivity in Iran's public hospitals in a 2 year period [13]. It was also shown that the teaching hospitals that were mainly affiliated with MOHME were suffering from technical inefficiency [14]. Other studies in different parts of Iran showed that the efficiency and productivity of hospitals after HSEP had a concerning status [15–17]. The aim of this study was to track hospital productivity before and after HSEP in Khuzestan province.

## Methods

### Study design

In this cross-sectional panel data study, the data were collected from 17 general and specialized hospitals of Ahvaz University of medical sciences, Khuzestan, Iran, during 2012–2016. Khuzestan province is one of the important provinces of Iran, which is rich in underground resources in such a way that a significant part of Iran’s gross domestic product is related to Khuzestan province [18]. This province is the fifth most populated province of Iran and has a high ethnic diversity. On the other hand, significant inequality in terms of health indicators has been reported from Khuzestan [19]. The data collection process in this study was retrospective and was used with the hospital information system for this purpose.

The data envelopment analysis (DEA) based Malmquist productivity index was used to measure productivity changes over time. DEA is a none-linear programming method designed to measure the relative efficiencies of a set of Decision-Making Units (DMU) such as hospitals (6). We selected the output-orientation approach and the variable returns to scale (VRS) model for data analysis. Since the assumption of constant returns to scale is appropriate only in certain scenarios, the assumption of variable returns to scale was used in this study [20]. The latter assumption seems more appropriate in the dynamic environment created by health system reforms. Many believe that public hospitals tend to maximize the output of available resources [20–22]. This situation becomes more tangible in the reforms of the health system, where hospitals are under pressure to increase the provision of health services. Therefore, the output orientation is more suitable than the input orientation for evaluating the hospital in this study. Input and output variables included hospital beds, the number of nurses, the number of patients admitted to hospitals, the mean hospital length of stay, and the bed turnover rate.

### Malmquist productivity index

We used the DEA-based Malmquist Productivity Index (MPI) to assess productivity changes over a 5 year period. The MPI takes different values to represent changes in productivity. When the MPI is equal to one, it indicates stagnation of productivity. Also, values larger and smaller than one indicate growth and decline in productivity, respectively [13]. The assessment of productivity changes over time along with its decomposition into efficiency changes and technology changes could be carried out using the so-called Malmquist index which was first introduced by Caves et al. based on the idea of Malmquist [23]. According to this approach, Total Factor Productivity (TFP) includes technological, managerial, and scale efficiency.

$${\text{TFP}}\, = \,{\text{TECH}}\, \times \,\left[ {{\text{PECH}}\, \times \,{\text{SECH}}} \right]$$

TECH is a measure of technical change in the hospital production technology, i.e. it measures the shift in technology use between the years *t* and *t* + 1. PECH and SECH stand for pure efficiency (managerial) and scale efficiency changes.

### Data analysis

The data were analyzed using DEAP V.2.1 software. Productivity changes were then analyzed using two models. In the first model, productivity changes were estimated for all of the hospitals (n = 17), and in the second, the 17 hospitals were categorized based on bed size (small, medium, and large hospitals) and type of hospitals (general hospital VS specialized hospital), and productivity changes were estimated for them.

## Results

Table 1 shows the (geometric) average change of the input-based MPI along with its decomposition into the components of efficiency change. In a 5 year period, the average changes in technical and scale efficiency were negative. However, technological efficiency had positive changes, and the MPI had low positive changes during 2013–2016 (0.13) as well. The comparison of the efficiency and productivity indicators before and after HSEP showed that EFFCH, PECH, and SECH had negative changes but TECHCH had positive changes after HSEP. The Malmquist Productivity Index (MPICH) had no change after the health sector evolution plan.

**Table 1:** Average change of hospital efficiency and productivity before and after HSEP* (2013–2016)

Indicators**	Before HSEP* (2013) (a)***	One-year after HSEP (2014)	Two-years after HSEP (2015)	Three-years after HSEP (2016)	Geometric mean of after HSEP (2014–2016) (b)	Deference (b-a)
EFFCH	1.06	1.07	0.75	0.85	0.89	−0.17
TECHCH	0.89	0.80	1.19	1.26	1.08	0.19
PECH	1.08	1.30	0.84	0.77	0.97	−0.11
SECH	0.98	0.83	0.89	1.11	0.94	−0.04
MPICH	0.94	0.86	0.89	1.07	0.94	0

^*^stands for HSEP

^**^refers to the estimated indicators in the first column

^***^refers to values of indicators that we entitled them as A score

According to Table 2, four hospitals out of 9 general ones (44.4%) had positive changes in the total productivity index after the HSEP. In addition, one o of the 4 multi-specialized hospitals (25%) had a positive productivity index change, and all of the specialized hospitals had positive productivity index changes as well.

**Table 2:** Productivity change of hospitals based on Malmquist index before and after HSEP (n = 17 hospitals)

Hospitals	Bed size	Type	Before HSEP (2013) (a)	One-year after HSEP (2014)	Two-years after HSEP (2015)	Three-years after HSEP (2016)	Geometric mean of after HSEP (2014–2016) (b)	Deference (b-a)
H1	526	Multi- specialized	0.75	1.03	0.97	0.96	0.98	0.23
H2	591	Multi- specialized	1.03	0.98	0.96	1.00	0.98	−0.05
H3	210	Multi- specialized	1.03	0.94	0.97	1.07	0.99	−0.03
H4	140	Specialized	0.89	0.96	0.82	0.92	0.90	0.02
H5	151	Specialized	1.05	0.96	0.99	1.30	1.08	0.04
H6	268	Multi- specialized	0.99	0.92	0.62	1.07	0.87	−0.12
H7	95	Specialized	0.79	0.88	0.88	0.93	0.90	0.11
H8	195	Specialized	0.93	0.94	1.66	1.31	1.30	0.37
H9	131	General	1.10	1.05	0.89	0.99	0.98	−0.12
H10	131	General	0.96	1.49	0.92	2.44	1.62	0.66
H11	122	General	1.07	0.96	0.67	0.86	0.83	−0.23
H12	116	General	1.14	0.82	0.65	0.67	0.71	−0.43
H13	132	General	1.05	1.04	0.91	0.84	0.93	−0.12
H14	88	General	0.93	0.93	1.00	0.89	0.94	0.01
H15	52	General	1.03	0.92	1.05	0.93	0.97	−0.06
H16	21	General	0.58	0.33	0.76	1.21	0.76	0.18
H17	23	General	0.89	0.32	0.81	1.73	0.95	0.06

Table 3 shows that after the HSEP, the mean technical efficiency change slightly decreased in the general hospitals. However, the mean technological and managerial efficiency increased. The mean scale efficiency change decreased and the MPI had no change. In the specialized hospitals, the mean technical efficiency changes decreased. On the other hand, the mean technological and managerial efficiency increased. The mean scale efficiency change decreased and the MPI had positive changes. In the multi-specialized hospitals, the mean technical efficiency changes decreased, the mean technological and managerial efficiency changes increased, and the mean scale efficiency change decreased. Finally, the MPI had slightly positive changes.

**Table 3:** Average change of efficiency and productivity based on type of hospitals before and after HSEP (2013–2016)

Indicators*	2013**			2014**			2015**			2016**		
	MSH	SH	GH	MSH	SH	GH	MSH	SH	GH	MSH	SH	GH
EFFCH	1	1	1.1	1	1	1.2	0.6	0.7	0.8	0.8	0.9	0.9
TECHCH	0.9	0.9	0.9	1	1	0.7	1.4	1.5	1	1.2	1.2	1.3
PECH	0.8	0.9	0.9	1.3	1.1	1.3	0.7	0.9	0.9	1	1.3	1.1
SECH	1.4	1.1	1.4	0.8	0.9	0.9	0.9	0.8	1	0.8	0.8	0.8
MPICH	0.9	0.9	1	1	0.9	0.9	0.9	1.1	0.9	1	1.1	1.2

Type of hospitals: *MSH* = multi-specialized hospital, *SH* = specialized hospital, *GH* = general hospital

^*^refers to the estimated indicators in the first column

^**^refers to values of indicators that we entitled them as A score

In Table 4, a summary of the efficiency and productivity changes is provided based on hospital size. After the HSEP, the mean technical efficiency changes decreased for all of the hospitals, but the mean technological efficiency changes increased significantly. The scale efficiency had negative and the MPCH had positive changes in all types of hospitals. According to Table 4, before and after the HSEP, the mean productivity change of small hospitals (< 100 beds) was lower than that of medium and large hospitals.

**Table 4:** Summary of the efficiency and productivity change based on hospitals’ size (2013–2016)

Indicators^*^	2013**			2014**			2015**			2016**		
	< 100	100–200	>200	< 100	100–200	>200	< 100	100–200	>200	<100	100–200	>200
EFFCH	1	1.1	1	1	1.3	1	0.9	0.7	0.6	0.9	0.9	0.8
TECHCH	0.8	0.9	0.9	0.7	0.9	1	1.1	1.3	1.3	1.3	1.3	1.2
PECH	0.8	0.9	0.8	1.1	1.3	1.3	0.9	0.9	0.6	1	1.2	1.1
SECH	1.3	1.3	1.4	0.9	0.9	0.8	1	0.9	1	0.9	0.8	0.8
MPICH	0.8	1	0.9	0.7	1	1	0.9	0.9	0.9	1.1	1.2	1

^*^refers to the estimated indicators in the first column

^**^refers to values of indicators that we entitled them as A score

## Discussion

This study was conducted with the aim of investigating the impact of recent reforms in Iran's health sector on the efficiency of public hospitals in Khuzestan province. The purpose of this study was to investigate the effect of recent reforms in Iran's health sector on the efficiency of public hospitals in Khuzestan province. This technique was also used to track the changes in efficiency and productivity of the studied hospitals over time before and after the healthcare reform or economic crisis in other studies [24, 25]. The results showed that after the ongoing HSEP, technical efficiency, pure technical efficiency, and scale efficiency had negative but technological efficiency had positive changes. Also, our findings showed that the productivity index has not changed after the implementation of this plan. This is in line with the former study that investigated the performance of the hospitals in Kerman province in Iran [17]. To increase the total productivity index, it is necessary to increase technical, scale, and pure efficiency as key components of the total productivity index. There is a variety of evidence on the impact of healthcare reforms on hospital productivity and efficiency. While some studies showed that healthcare reforms might increase the efficiency and productivity of these health service providers [26–29], some others showed that the number of efficient hospitals decreased after reforms [30]. Our finding is in line with the findings of a former study in China that showed the productivity of public hospitals did not experience significant fluctuations [31]. It seems that healthcare reforms did not have the same effect on the productivity of hospitals at the sub-national level. Different studies in China reported different effects of healthcare reforms on the performance of hospitals [26, 31, 32]. The heterogeneous effect of HSEP on the performance of hospitals was also reported in different parts of Iran [17, 33]. Since HSEP increased the demand for inpatient services in Iran's public hospitals, it seems that the lack of change in efficiency and productivity is a sign of the good performance of these hospitals in Khuzestan. However, this could not satisfy healthcare policy-makers. Efforts to reduce OOP cannot simply be considered a health care reform. Hence, the current HSEP should be amended through the introduction of cost containment requirements in the future to address potential inefficiencies in the health system, particularly with a focus on the inpatient sector. In addition, hospitals in different regions of Iran may have different requirements that affect their productivity. Thus, planned decentralization of the health sector may increase hospital productivity at the subnational levels [34].

Hospitals that do not operate efficiently cannot remove excess inputs without changes in the output levels [13], and this has a negative effect on their productivity. According to the findings of a previous study, nearly 40% of Iran's hospitals provide services inefficiently [35]. Since there is a close relationship between efficiency and productivity, it can be said that there is a lack of productivity in Iranian hospitals. Inefficiency in providing inpatient services is not unique to Iran's health system. Kirigia et al. reported that 68% of public hospitals were technically efficient and only 42% had scale efficiency in Eritrea. They suggested that inefficient hospitals either increased their outputs by outpatient visits and hospital discharges, or transferred the excess human resources and beds to other healthcare facilities [36]. In our study, only 44.4% of general hospitals, 25% of multi-specialized hospitals, and all specialized hospitals had positive productivity changes after implementing HSEP. Therefore, other inefficient hospitals should manage their inputs and outputs based on reference hospitals (productive hospitals). There is no solid evidence about the efficiency of hospitals and the expertise of these organizations.An Iranian study showed that the efficiency score of specialized hospital was higher than multi-specialized and general ones [37]. The results of another study in Yazd province indicate that there is no significant difference between the efficiency scores of general and specialized hospitals [38]. The results of the present study showed that after HSEP, the mean scores of productivity change in small (< 100 beds), medium (100–200 beds), and large hospitals (> 200 beds) were 0.9, 1.03, and 0.96, respectively. Therefore, the productivity score of small hospitals was lower than medium and large ones. This could be attributed to the lower economy of scale in these hospitals. Different studies assessed the impact of hospital size on efficiency and productivity changes. One study found consistent evidence of economies of scale for hospitals with 200 to 300 beds, as well as diseconomies of scale in hospitals with less than 200 and more than 600 beds [39]. Cheng et al. stated that hospitals with > 618 beds had technical inefficiency [40]. The results of these studies are in accordance with the results of the present research. Very Small and large hospitals are generally nonproductive and inefficient [41].

In this study, the total productivity change scores were appropriate (0.7–1.4). Torabipour et al. carried out a similar study in Iranian public hospitals and showed that the mean productivity score of hospitals was 1.024 [13]. Chang et al. reported an increase in the productivity index of thirty regional hospitals in Taiwan (0.874) [13]. To improve the total productivity index of hospitals, economies of scale methods including adjusting hospital beds, increasing outputs, and reducing hospital length of stay are appropriate [13, 42]. Economies of scale and scope are affected by hospital size [41].

### Limitations of Study

This study had some limitations that must be declared. First, our study had a small sample size, which may make many hospitals efficient by default. Second, fiscal data were not available completely and accurately in the studied hospitals; therefore, calculating some efficiency indexes might be associated with problems. Last, since there was no accurate data about all hospitals’ workforce (doctors, nurses, clinical officers, laboratory technicians, and anesthetic officers), we considered only the number of nurses as one of the inputs.

## Conclusions

DEA-based productivity analysis is an applied and useful technique to analyze hospital productivity and performance. We found that the average hospital productivity score decreased from 2013–2016, but the change in total productivity before and after HSEP was unchanged. No change in productivity along with the increase in public inpatient services could be considered as the good performance of public hospitals in Khuzestan. However, while technological efficiency had positive changes, other efficiency indexes were negative, and this should not satisfy healthcare policy makers. Hence, healthcare authorities at national and sub-national levels must enrich this ongoing plan with cost containment requirements. It seems that adjusting the hospital beds, increasing the output and reducing the length of stay in the hospital can increase productivity in the public hospitals of Khuzestan.

## Data Availability

The datasets used during the current study are available from the corresponding author on reasonable request.

## References

[1] Putera I (2017). Redefining health: implication for value-based healthcare reform. Cureus.

[2] Jiang S, Min R, Fang P-q (2017). The impact of healthcare reform on the efficiency of public county hospitals in China. BMC Health Serv Res.

[3] Bastani P, Vatankhah S, Salehi M (2013). Performance ratio analysis: a national study on Iranian hospitals affiliated to ministry of health and medical education. Iran J Public Health.

[4] Hajizadeh M, Nghiem HS (2011). Out-of-pocket expenditures for hospital care in Iran: who is at risk of incurring catastrophic payments?. Int J Health Care Finance Econ.

[5] Kameli M, Behtaj F, Vahedi-barzaki A, Lotfi-golmisheh F: Statistical report of hospitals and information system. Tehran: Office of Hospital Management and Clinical Services Excellence-Ministery of Health and Medical Education; 2016. (in Persian).

[6] Moradi-Lakeh M, Vosoogh-Moghaddam A (2015). Health sector evolution plan in Iran; equity and sustainability concerns. Int J Health Policy Manag.

[7] Vahedi S, Yazdi-Feyzabadi V, Amini-Rarani M, Mohammadbeigi A, Khosravi A, Rezapour A (2020). Tracking socio-economic inequalities in healthcare utilization in Iran: a repeated cross-sectional analysis. BMC Public Health.

[8] Bahadori M, Izadi AR, Ghardashi F, Ravangard R, Hosseini SM (2016). The evaluation of hospital performance in Iran: a systematic review article. Iran J Public Health.

[9] Xenos P, Yfantopoulos J, Nektarios M, Polyzos N, Tinios P, Constantopoulos A (2017). Efficiency and productivity assessment of public hospitals in Greece during the crisis period 2009–2012. Cost Eff Resour Alloc.

[10] De Nicola A, Gitto S, Mancuso P, Valdmanis V (2014). Healthcare reform in Italy: an analysis of efficiency based on nonparametric methods. Int J Health Plann Manage.

[11] Linna M, Häkkinen U, Peltola M, Magnussen J, Anthun KS, Kittelsen S, Roed A, Olsen K, Medin E, Rehnberg C (2010). Measuring cost efficiency in the Nordic Hospitals—a cross-sectional comparison of public hospitals in 2002. Health Care Manag Sci.

[12] Kiadaliri AA, Jafari M, Gerdtham U-G (2013). Frontier-based techniques in measuring hospital efficiency in Iran: a systematic review and meta-regression analysis. BMC Health Serv Res.

[13] Torabipour A, Najarzadeh M, Mohammad A, Farzianpour F, Ghasemzadeh R (2014). Hospitals productivity measurement using data envelopment analysis technique. Iran J Public Health.

[14] Goudarzi R, Pourreza A, Shokoohi M, Askari R, Mahdavi M, Moghri J (2014). Technical efficiency of teaching hospitals in Iran: the use of stochastic frontier analysis, 1999–2011. Int J Health Policy Manag.

[15] Pirani N, Zahiri M, Engali KA, Torabipour A (2018). Hospital efficiency measurement before and after health sector evolution plan in Southwest of Iran: a DEA-panel data study. Acta Inform Med.

[16] Kakemam E, Dargahi H (2019). The health sector evolution plan and the technical efficiency of public hospitals in Iran. Iran J Public Health.

[17] Goudarzi R, Tasavon Gholamhoseini M, Noori Hekmat S, YousefZadeh S, Amini S (2021). The effect of Iran’s health transformation plan on hospital performance: Kerman province. PLoS ONE.

[18] Razzaghi A, Soori H, Kavousi A, Abadi A, Khosravi A (2019). Factors with the highest impact on road traffic deaths in Iran; an ecological study. Arch Acad Emerg Med.

[19] Ahangari A, Baghlani M (2016). The evaluation of development degree in cities of Khuzestan in terms of health care indicators in 2008 and 2013. J Econ Reg Dev.

[20] Kucuk A, Ozsoy VS, Balkan D (2020). Assessment of technical efficiency of public hospitals in Turkey. Eur J Public Health.

[21] Al-Hanawi MK, Makuta IF (2022). Changes in productivity in healthcare services in the Kingdom of Saudi Arabia. Cost Eff Resour Alloc.

[22] Vankova I, Vrabkova I (2022). Productivity analysis of regional-level hospital care in the Czech republic and Slovak Republic. BMC Health Serv Res.

[23] Flokou A, Aletras V, Niakas D (2017). A window-DEA based efficiency evaluation of the public hospital sector in Greece during the 5-year economic crisis. PLoS ONE.

[24] Şahin B, İlgün G (2019). Assessment of the impact of public hospital associations (PHAs) on the efficiency of hospitals under the ministry of health in Turkey with data envelopment analysis. Health Care Manag Sci.

[25] Mitropoulos P, Mitropoulos I, Karanikas H, Polyzos N (2018). The impact of economic crisis on the Greek hospitals' productivity. Int J Health Plann Manage.

[26] Wang ML, Fang HQ, Tao HB, Cheng ZH, Lin XJ, Cai M, Xu C, Jiang S (2017). Bootstrapping data envelopment analysis of efficiency and productivity of county public hospitals in Eastern, Central, and Western China after the public hospital reform. J Huazhong Univ Sci Technol Med sci.

[27] Sommersguter-Reichmann M (2000). The impact of the Austrian hospital financing reform on hospital productivity: empirical evidence on efficiency and technology changes using a non-parametric input-based Malmquist approach. Health Care Manag Sci.

[28] Pham TL (2011). Efficiency and productivity of hospitals in Vietnam. J Health Organ Manag.

[29] Cheng Z, Tao H, Cai M, Lin H, Lin X, Shu Q, Zhang RN (2015). Technical efficiency and productivity of Chinese county hospitals: an exploratory study in Henan province, China. BMJ Open.

[30] van Ineveld M, van Oostrum J, Vermeulen R, Steenhoek A, van de Klundert J (2016). Productivity and quality of Dutch hospitals during system reform. Health Care Manag Sci.

[31] Du J, Cui S, Gao H (2020). Assessing productivity development of public hospitals: a case study of Shanghai, China. Int J Env Res Public Health.

[32] Li H, Dong S, Liu T (2014). Relative efficiency and productivity: a preliminary exploration of public hospitals in Beijing China. BMC Health Serv Res.

[33] Bastani P, Lotfi F, Moradi M, Ahmadzadeh M (2016). The performance analysis of teaching hospitals affiliated with shiraz university of medical sciences before and after health system reform plan using pabon lasso model. J Rafsanjan Univ Med Sci.

[34] Abimbola S, Baatiema L, Bigdeli M (2019). The impacts of decentralization on health system equity, efficiency and resilience: a realist synthesis of the evidence. Health Policy Plan.

[35] Shahhoseini R, Tofighi S, Jaafaripooyan E, Safiaryan R (2011). Efficiency measurement in developing countries: application of data envelopment analysis for Iranian hospitals. Health Serv Manage Res.

[36] Carey K, Burgess JF, Young GJ (2008). Specialty and full-service hospitals: a comparative cost analysis. Health Serv Res.

[37] Azar A, Khatir MV, Baerz AM, Yeganeh YH (2013). Evaluation of hospital efficiency by data envelopment analysis: Tehran University of Medical Sciences: 2009–2011. J Health Adm.

[38] Roohollah A, Reza G, Hossein F, Bahareh Z, Arefeh DT (2012). Efficiency appraisal of Yazd University of medical science hospitals by quantitative approach data envelopment analysis (DEA). Payavard Salamat.

[39] Giancotti M, Guglielmo A, Mauro M (2017). Efficiency and optimal size of hospitals: results of a systematic search. PLoS ONE.

[40] Cheng Z, Tao H, Cai M, Lin H, Lin X, Shu Q (2015). Zhang R-n: Technical efficiency and productivity of Chinese county hospitals: an exploratory study in Henan province, China. BMJ Open.

[41] Weaver M, Deolalikar A (2004). Economies of scale and scope in Vietnamese hospitals. Soc Sci Med.

[42] Mujasi PN, Asbu EZ, Puig-Junoy J (2016). How efficient are referral hospitals in Uganda? A data envelopment analysis and tobit regression approach. BMC Health Serv Res.

